# Atypical Presentation of a Sequestered Posterolateral Disc Fragment

**DOI:** 10.7759/cureus.502

**Published:** 2016-02-20

**Authors:** Olaide Ajayi, Alireza Shoakazemi, R. Shane Tubbs, Marc Moisi, Steven Rostad, David W. Newell

**Affiliations:** 1 Department of Neurosurgery, Loma Linda University; 2 Neurosurgery, Swedish Neuroscience Institute; 3 Neurosurgery, Seattle Science Foundation; 4 Cellnetix Pathology, CellNetix; 5 Swedish Neuroscience Institute

**Keywords:** lumbar disc, spine tumor

## Abstract

Sequestered disc fragments typically occur ventrally but can also migrate dorsally or intradurally. At times, atypical disc herniations can be misinterpreted on imaging as other lesions, such as neoplasms, hematomas, or abscesses. We present an uncommon case of a patient presenting with cauda equina syndrome secondary to an enhancing sequestered disc fragment mimicking a tumor.

## Introduction

Sequestered intervertebral disc fragments have the potential to migrate both intradurally and extradurally within the spinal canal. There are no particular clinical features allowing for a clear differentiation between patients with atypical disc herniations and those with tumors [1]. Free disc fragments were previously only identified during surgery but are still frequently misinterpreted as neoplastic masses, even after the introduction of magnetic resonance imaging (MRI) [1-3]. This is because the imaging characteristics of sequestered disc fragments may mimic known characteristics of extramedullary (intra- and extradural) lesions, including neoplasms and other benign epidural lesions (such as synovial cysts, hematomas, and abscesses), further complicating preoperative diagnosis based on imaging findings [1, 3].

We present an uncommon case of a cauda equina syndrome secondary to a large extradural ring-enhancing sequestered disc fragment, mimicking a tumor.

## Case presentation

A 65-year-old female presented with a one-month history of progressively worsening lower back pain radiating down her bilateral lower extremities in the L5 distribution, bilateral lower extremity weakness, bilateral anterior thigh numbness, and difficulty ambulating for two weeks. She denied any antecedent trauma or strenuous activity and did not report any bowel or bladder incontinence. Her medical history was significant for obesity and pulmonary fibrosis, and negative for neoplasms, recent infections, or recent surgeries. She was afebrile with normal inflammatory markers, including white blood cell count, C-reactive protein, and sedimentation rates.

Her neurological examination was intact in the upper extremities, but she had lower extremity paresis and reduced sensation to light touch on both anterior thigh areas with hyporeflexic knee and ankle jerk reflexes (Table 1). Examination of anal tone was unremarkable.

**Table 1:** Preoperative Neurological Evaluation of Lower Limbs Based on Medical Research Council (MRC) Scores

Movement	Right Lower Limb	Left Lower Limb
Hip Flexion	3/5	2/5
Knee Extension	3/5	2/5
Plantar Flexion	3/5	2/5
Dorsiflexion	3/5	1/5
Great Toe Extension	3/5	1/5

Informed patient consent was obtained. No identifying patient information is disclosed in this report.

### Radiologic findings

**Figure 1:**
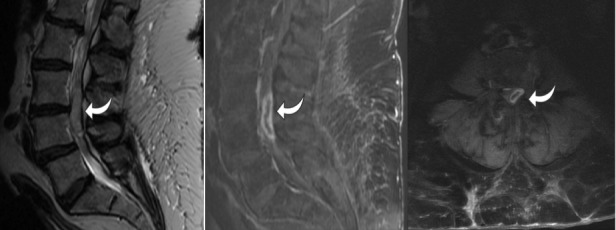
Preoperative Lumbar Spine MRI T2 sagittal, T1 sagittal contrast-enhanced, and T1 axial contrast-enhanced MRI of the lumbar spine showing a 30 mm × 5 mm × 7 mm L3-4 extradural mass that is mildly T2 hyperintense and T1 hypointense with a thick rim of contrast enhancement (arrow)

MRI scan of the lumbar spine with and without contrast showed a left 30 mm × 5 mm × 7 mm L3-4 extradural mass that was mildly T2 hyperintense and T1 hypointense with a thick rim of contrast enhancement (Figure 1). The differential diagnosis included schwannoma, neurofibroma, extradural meningioma, and abscess.

### Operative management

The patient was urgently taken to the operating room where an L3-4 laminectomy was performed for resection of an assumed intradural tumor. Once the laminectomy was performed, a firm left-sided posterolateral extradural rubbery pink/tan mass was encountered. A gross total resection was achieved and the specimen was sent for pathological examination. 

### Pathology

**Figure 2:**
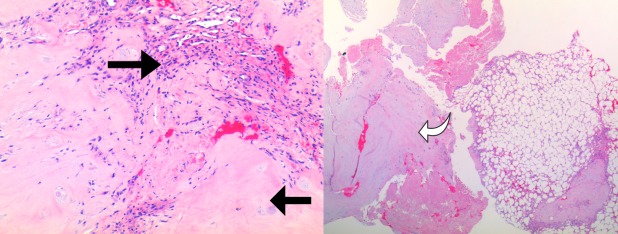
Pathology The specimen contains mild patchy lymphoplasmacytic inflammation (arrow pointing to the right), focal degenerate acellular material (arrow pointing to the left), and fibrocartilage with degenerative features (curved arrow)

The specimen consisted of fibrocartilage with degenerative features (i.e., increased chondrocyte density, mucoid degeneration, vascular proliferation, and granular change). Focally, the disc material was surrounded by mature adipose tissue consistent with soft tissue migration. Mild patchy lymphoplasmacytic inflammation was present. No atypia or mitotic figures were noted in the disc material. Focally, degenerate acellular material was noted and tested positive for amyloid on Congo red stain and was considered degenerative in origin. Immunostains to S100, glial fibrillary acidic protein, low molecular weight keratins, and brachyury were negative for myxopapillary ependymoma and chordoma. No active inflammation was noted. There was no evidence of neoplasm. The histologic features were characteristic of intervertebral disc tissue with soft tissue migration and degeneration (Figure 2).

### Postoperative management and follow-up

By postoperative day 1, the patient’s neurological examination had significantly improved. She was discharged to the rehabilitation facility on postoperative day 4 with 4+/5 strength in her right lower extremity and 4/5 strength in her left lower extremity in all muscle groups. Her anterior thigh numbness completely resolved by postoperative day 4. 

## Discussion

In 1973, Lombardi first reported a case of posterior epidural migration of a lumbar disc fragment and described the lesion as a “posterior rotation of annulus fibrosus” [4-5]. Based on a retrospective review of MR images of 3,000 patients with a histological diagnosis of herniated disc, Carvi y Nievas, et al. identified a 0.4% (11 patients) incidence of unusual sequestered disc fragments that were mistaken for other space-occupying lesions. The fragment had migrated to the posterior spinal space in eight cases, into the dural sac in three cases, and was distant from the original disc space in three cases [1].

The rarity of posterolateral disc fragments is thought to be secondary to the presence of various anatomical barriers that limit their migration. One such barrier is the sagittal midline septum (septum posticum), which spans the space between the vertebral body and the posterior longitudinal ligaments, thus preventing the movement of the herniated disc fragment across the midline [4]. Another barrier is the peridural or lateral membrane, which attaches posterolaterally to the free edge of the posterior longitudinal ligament, thus limiting the posterior migration of a free disc fragment [4].

Despite the improved anatomical definition with MRI, some disc fragments can still be mistaken for other epidural and intradural benign lesions or neoplasms. The absence of accompanying disc space protrusions on MRI also carries a particular risk for a misdiagnosis [1]. Disc fragments can show different patterns of contrast enhancement and further complicate the preoperative diagnosis. Peripheral enhancement of the disc fragment on contrast-enhanced imaging studies is due to the development of an inflammatory response with vascular granulation tissue on the periphery of the disc. The presence of peripheral rim enhancement and recognizing the similarity between the signal intensity of the mass and the intervertebral disc are helpful clues to correctly diagnose a sequestered posterolateral disc fragment.

## Conclusions

Cases of large sequestered or posterolateral disc fragments may present with a significant compression of neural elements and may have imaging characteristics that mimic neoplasms or other benign extradural, extramedullary, or intradural lesions. Intraoperative findings could also be misleading in some cases. Therefore, the differential diagnosis of a rim or heterogeneously enhancing extradural, extramedullary lesion should include a posterolateral intervertebral disc fragment. The correct preoperative diagnosis of these lesions greatly depends on the identification of important imaging findings, which include a comparison of MRI signal intensity between the mass and the intervertebral disc as well as peripheral rim enhancement on MR imaging.
